# “Physiological and pathological responses to hypoxia and high altitude, volume IV.” Mechanisms of reduction in V̇O_2_max in hypoxia/at altitude

**DOI:** 10.3389/fphys.2026.1970453

**Published:** 2026-10-26

**Authors:** Peter D. Wagner

**Affiliations:** Division of Pulmonary, Critical Care, and Sleep Medicine and Physiology, Department of Medicine, University of California, San Diego, San Diego, CA, United States

**Keywords:** altitude, cardiac output, exercise, hemoglobin, hypoxia, lung O_2_ diffusion, muscle O_2_ diffusion, V̇O_2_max

## Abstract

On Mt. Everest, maximal O_2_ uptake ( $\dot{\text{V}}{\text{O}}_{2}\max$) in fit young subjects, around 4-5l/min at sea level, falls to only ~1l/min. The drop in PIO_2_ itself as barometric pressure decreases with altitude would theoretically explain about half of that reduction. Limitations in the O_2_ transport system (lungs, heart/circulation and muscles) increase with altitude and account for the rest. Here the O_2_ transport system is analyzed using both physiological theory and published experimental data to show that O_2_ diffusion limitation in the lungs and muscles appears to be the primary basis for this, as the laws of diffusion would dictate, based on ever smaller O_2_ diffusion gradients with altitude. On the other hand, changes in the convective processes in O_2_ transport (ventilation and blood flow) appear not to explain most of the fall in $\dot{\text{V}}{\text{O}}_{2}\max$ with altitude. Maximal ventilation is well-maintained at sea level values despite the low $\dot{\text{V}}{\text{O}}_{2}\max$, causing arterial PCO_2_ to fall to single digit values. While maximal cardiac output falls with altitude, multidisciplinary studies show preserved cardiac function even at the Everest summit. Additionally, both theoretical calculations and experimental data suggest that lower maximal cardiac output is not likely a major factor in the reduction in $\dot{\text{V}}{\text{O}}_{2}\max$. The increase in hemoglobin concentration with time at altitude has also been shown not to affect $\dot{\text{V}}{\text{O}}_{2}\max$. The ongoing debate about the role of the cardiovascular system in limiting $\dot{\text{V}}{\text{O}}_{2}$ at altitude will likely continue until a study can be devised that restores cardiac output at altitude to sea level norms.

## Introduction

It is well known that maximal exercise falls as altitude is gained. Much has been made of the finding that under conditions equivalent to the summit of Mt. Everest, $\dot{\text{V}}{\text{O}}_{2}\max$ of a fit healthy climber, generally about 4 l/min at sea level, is reduced to barely above 1 l/min (West et al., 1983; Cymerman et al., 1989). This is just enough to support moderately paced walking on the level. While this must of course be fundamentally the result of reduced inhaled O_2_ secondary to lower barometric pressure, the question of how that loss of pressure explains the fall in $\dot{\text{V}}{\text{O}}_{2}\max$ in physiological terms needs answering, and that is the goal of this Perspective. Several key observations need to be presented to frame the issue.

First, as barometric pressure falls, so too does inspired PO_2_. This in turn reduces alveolar and arterial PO_2_ and O_2_ concentration, and therefore also arterial O_2_ delivery (blood flow x arterial O_2_ concentration) to the muscles. However, this direct, physical consequence of altitude explains only about half the reduction in $\dot{\text{V}}{\text{O}}_{2}\max$ from sea level to the Everest summit. This will be detailed in the analysis to follow. The remainder is attributable to the combined effects of three main barriers to O_2_ transport in the body: barriers imposed by A) impaired pulmonary gas exchange B) reduced maximal cardiac output and C) incomplete O_2_ extraction by the muscles. Theoretical calculations presented in this article suggest roughly equal contributions from these three barriers, but such predictions depend on assumptions about how the several steps of the O_2_ transport pathway would interact quantitatively as they change with altitude.

Second, breathing hyperoxic gas at sea level results in a small but measurable increase in $\dot{\text{V}}{\text{O}}_{2}\max$ (Peltonen et al, 2001; Prieur et al., 2002). In addition, after acclimatization to, and while still at, altitude, $\dot{\text{V}}{\text{O}}_{2}\max$ is immediately restored to sea level values by breathing hyperoxic gas (Calbet et al, 2003). These studies show that $\dot{\text{V}}{\text{O}}_{2}\max$ in healthy fit adults, whether at sea level or altitude, is not limited by mitochondrial capacity to use O_2_, but rather by the ability to deliver O_2_ from the air to the mitochondria.

Third, it is well known that pulmonary gas exchange suffers at altitude – more so than at sea level. Both increased ventilation/perfusion inequality and, especially, alveolar-capillary diffusion limitation develop at altitude (Wagner et al, 1986; West, 1990). Together these reduce arterial O_2_ tension, saturation and concentration, and as a result, also lower O_2_ delivery to the muscles. This is therefore a barrier to maintaining $\dot{\text{V}}{\text{O}}_{2}\max$ at altitude.

A fourth important observation is that maximal cardiac output falls with acclimatization to altitude as mentioned above (Grover et al., 1986; Sutton et al., 1988; Bebout et al., 1989; Wolfel and Levine, 2001). Other factors unchanged, reduced maximal cardiac output will lower arterial O_2_ delivery and hence $\dot{\text{V}}{\text{O}}_{2}\max$.

A fifth critical observation is that at both sea level and altitude the venous blood returning from exercising muscle to the lungs is not fully depleted of O_2_ even at/near $\dot{\text{V}}{\text{O}}_{2}\max$ (Saltin et al., 1968; Sutton et al, 1988; Roca et al., 1989; Agustí et al., 1994), with venous blood still containing some 10-30% as much O_2_ as in the arterial blood. This observation, combined with the above-mentioned increase in $\dot{\text{V}}{\text{O}}_{2}\max$ in hyperoxia, indicate a barrier to the muscles extracting and using all of the O_2_ they are supplied and are capable of using.

Additional relevant findings that play into this Perspective are that as lowlanders reside at and acclimatize to altitude, ventilation increases and polycythemia develops. These responses work to compensate the persisting arterial hypoxemia in terms of oxygen carriage, and may even return total O_2_ delivery (the product of blood flow and arterial O_2_ concentration) to (near) sea level values (Calbet et al., 2003). However, returning O_2_ delivery to normal in this way has only a very small effect on $\dot{\text{V}}{\text{O}}_{2}\max$, which remains, remarkably, considerably lower than at sea level (Calbet et al, 2003). In addition, after acclimatization, acutely (and isovolumically) reducing the high hemoglobin concentration to sea level values while still at altitude does not reduce $\dot{\text{V}}{\text{O}}_{2}\max$ beyond the value found at the same altitude prior to isovolumic blood removal (Calbet et al., 2002). These at first sight perplexing observations need to be accounted for in analyzing the fall in $\dot{\text{V}}{\text{O}}_{2}\max$ with altitude.

Taking all of these findings together shows that $\dot{\text{V}}{\text{O}}_{2}\max$ does not fall with altitude simply because there is less O_2_ in the inhaled air. There are barriers within the body to getting O_2_ from the air to the mitochondria, barriers that contribute to further reduce $\dot{\text{V}}{\text{O}}_{2}\max$ at both sea level and altitude. The purpose of this article is to integrate the above findings and analyze their relative importance to $\dot{\text{V}}{\text{O}}_{2}\max$ limitation, from both theoretical calculations and experimental findings.

## Analysis

To approach these questions, the O_2_ transport system that conveys O_2_ from the air to the muscle mitochondria to produce the energy for muscle contraction must be appreciated. There are several, well-established, distinct and sequential steps involved in moving O_2_ to the mitochondria and utilizing that O_2_ (Weibel, 1984), and they are:

1. Pulmonary ventilation.
2. Alveolar-pulmonary capillary diffusion.
3. Perfusive transport in the arterial blood from lungs to muscle.
4. Muscle microvascular-muscle mitochondrial diffusion.
5. Mitochondrial respiration via oxidative phosphorylation.

The question becomes: How does hypoxia/altitude affect O_2_ transport through each of these steps? Stated differently, which one - or more - of these 5 steps is responsible for the additional reduction in $\dot{\text{V}}{\text{O}}_{2}\max$ beyond that dictated by the fall in PIO_2_ as altitude is gained? The purpose of this article may be restated as addressing that question.

To do this, the fundamental principles of O_2_ transport need to be understood. The key point is to underscore that steps 1 and 3 above involve *convective* transport processes while steps 2 and 4 are based on *diffusive* transport processes. In the convective O_2_ transport steps, it is *concentration* of O_2_ (be it in alveolar gas or blood) that is relevant to determining O_2_ transport rather than O_2_ partial pressure (in particular because the O_2_ dissociation curve is non-linear). However, the diffusive transport steps - in gas, blood and tissues - are determined by O_2_ *partial pressures* and not by O_2_ concentrations. This fundamental physical principle will become the key to understanding how altitude reduces $\dot{\text{V}}{\text{O}}_{2}\max$ beyond the obvious reduction in inspired PO_2_.

The four convective and diffusive steps work as a system to deliver O_2_ to the mitochondria, which use O_2_ through oxidative phosphorylation to produce the energy for muscle contraction, depicted as step 5. The oxidative phosphorylation process follows Michaelis-Menten kinetics based on the law of mass action, meaning that mitochondrial O_2_ utilization ( $\dot{\text{V}}{\text{O}}_{2}$) is a hyperbolic function of mitochondrial PO_2_ (Gnaiger, 2003). That said, mitochondrial PO_2_ is it itself determined by the relationship between O_2_ supply (via the first four steps above) and O_2_ utilization (step five). Mitochondrial PO_2_ is thus at the same time both a driver of $\dot{\text{V}}{\text{O}}_{2}$ and the result of the balance between O_2_ availability and use.

It is logical to think of the 5 transport steps as occurring in a sequence in the order laid out above. This is because if one were to follow the journey undertaken by a single O_2_ molecule, it begins with inhalation from the air by ventilation, proceeds to alveolar-capillary diffusion, then to circulatory transport from lungs to muscle, then to diffusion from microvessels to mitochondria and finally into oxidative phosphorylation where it ends its journey by conversion to CO_2_ after producing the high energy molecules that fuel muscle contraction. This well-known sequence reminds one of a bucket brigade, where the above 5 steps represent 5 persons in the bucket brigade and each hands a bucket of O_2_ to the next in line in sequence.

While anatomically correct, this functional sequence does not address the critical fact that each of the five steps is affected by the functional state of each of the other four. It is not just the way in which altitude affects each step, but how altitude also affects their interactions that must be included in the thinking about how altitude reduces maximal $\dot{\text{V}}{\text{O}}_{2}$ and thus exercise capacity. As the discussion proceeds, we will review not only the function of each of the 5 steps, and how they each might change with altitude, but also their interactions, as fundamental to understanding the loss of overall exercise capacity with altitude.

Fortunately, the system for O_2_ transport can be understood quantitatively by applying the basic principle of mass conservation (of O_2_) at every step: No O_2_ is “spilled” from the “bucket” as it is passed through the five steps. In other words, the mass flow rate of O_2_ is the same through each of the steps. This principle allows the writing of a specific mass conservation equation for O_2_ movement through each step (Cano et al., 2013).

## Mass conservation and the five steps in oxygen transport

Step 1: Pulmonary ventilation is the essentially convective process of moving inspired air down the airways from the environment to the alveoli (as for water through a hose), and then moving alveolar gas back from the alveoli to the environment. Convective flow is of course greater during exercise than at rest as both tidal volume and respiratory frequency increase. The convective process of ventilation is powered by respiratory muscle contraction involving the diaphragm, intercostal, abdominal and accessory muscles. Conveniently, one simple mass conservation equation describes the ventilatory transport of O_2_ between air and the alveoli. Abbreviating O2 concentration to [O_2_], this equation is simply:


$$O_{2}\;taken\;up=ventilation\times inspired\left[O_{2}\right]-ventilation\times expired\left[O_{2}\right]$$

This equation implies a steady state of respiration and metabolism, which is a requirement throughout this analysis at every step. Even then, inspiratory ventilation (volume of air inhaled/minute) equals the expiratory ventilation (volume of gas exhaled/minute) only if the volume of O_2_ taken up ( $\dot{\text{V}}{\text{O}}_{2}$) equals the volume of CO_2_ breathed out ( $\dot{\text{V}}{\text{CO}}_{2}$). While this is commonly not the case, the difference between $\dot{\text{V}}{\text{O}}_{2}$ and $\dot{\text{V}}{\text{CO}}_{2}$ is almost always 1% or less of the total ventilation (V̇E), and therefore can here be set aside to simplify the presentation. Thus the above relationship can be written:

(1) $$\dot{V}{\text{O}}_{2}=\dot{\text{V}}\text{E}\times \left[{\text{FIO}}_{2}-{\text{FEO}}_{2}\right]=\dot{\text{V}}\text{A}\times \left[{\text{FIO}}_{2}-{\text{FAO}}_{2}\right]$$

where FIO_2_ is inspired [O_2_], FEO_2_ is mixed expired [O_2_], and FAO_2_ is mixed alveolar [O_2_]; V̇A is alveolar ventilation (that is, V̇E less anatomic airway ventilation). Note that O_2_ partial pressure, PO_2_, (be it PIO_2_, PEO_2_ or PAO_2_) = FO_2_ x (PB – SVP) where FO_2_ is fractional O_2_concentration, PB is barometric pressure and SVP is saturated water vapor pressure. With altitude, FIO_2_ and SVP do not change, but PIO_2_ falls because of the corresponding reduction in PB.

Step 2: Alveolar-capillary is a passive process whereby net movement of O_2_ molecules occurs from a region of higher O_2_ partial pressure (PO_2_) (in alveolar gas, PAO_2_) to a region of lower PO_2_ (in pulmonary capillary blood, PcapO_2_). Diffusion is random movement of molecules fueled by their thermal kinetic energy. While a directionally random process, it is based on molecular collisions, which are innately more frequent when PO_2_ is high than when it is low. In the lung, O_2_ diffusion thus causes net movement of O_2_ molecules from the high PO_2_ gas phase into the lower PO_2_ mixed venous blood entering the alveolar capillaries. This involves movement through the alveolar blood-gas barrier in which O_2_ dissolves and moves to reach the capillary plasma, where it remains dissolved. From there O_2_ diffuses into red cells and rapidly binds with Hb, the principal way in which O_2_ is moved through the vasculature. This diffusive process also obeys mass conservation: all O_2_ molecules moving across the blood gas barrier end up in the blood, and none are lost in transit. Because PcapO_2_ is low at the start of the lung capillary and rises toward PAO_2_ as the red cell flows though the alveolar vessels and diffusion takes place, mass O_2_ movement from gas to blood is faster early in the capillary and slows progressively as the red cell moves along the capillary. To quantify this process therefore requires a differential equation to answer the question “what is the PcapO_2_ at the end of the capillary as the red cell moves into the pulmonary veins?” The differential equation is quite straightforward:


$$\delta \dot{V}O_{2}\left(t\right)=DL\times \left[PAO_{2}-PcapO_{2}\left(t\right)\right]\times \delta t$$

This says that the small amount of O_2_ ( $\delta \dot{\text{V}}{\text{O}}_{2}$) diffused across the blood gas barrier in a small time interval δt at any point in time (t) along the capillary is the product of the lung diffusing capacity (DL) and the PO_2_ difference between alveolar gas (PAO_2_) and capillary blood (PcapO_2_(t)) at that same time point. In this equation, which expresses Fick’s law of diffusion, DL and PAO_2_ are considered constant in time and only PcapO_2_ changes with time - from mixed venous PO_2_ at the red cell’s entry into the pulmonary capillary to what is termed endcapillary PO_2_, the PO_2_ with which the red cell exits the capillary and enters the pulmonary vein. DL is considered as reflecting the combination of two sequential processes: a) diffusion of O_2_ from alveolar gas to capillary plasma and into the red cell, and b) the ensuing chemical binding of O_2_ to Hb inside the red cells. For simplicity, DL is here taken to be a single variable, but it actually has these two components (Roughton and Forster, 1957).

If one simplifies the calculation by assuming that the O_2_Hb dissociation curve is linear with a slope “β”, integration of the above differential equation yields this result (Piiper and Scheid, 1981).

(2) $$PAO_{2}-PecO_{2}=\left(PAO_{2}-PvO_{2}\right)\times \exp\left[-DL/\left(\beta \times \dot{Q}\right)\right]$$

PecO_2_ is the outcome variable of interest, being the PO_2_ in the capillary at the end of its alveolar transit. PvO_2_ is the starting PO_2_ in the red cell returning from the tissues as it enters the lungs (ie, the PO_2_ of mixed venous blood); and $\dot{\text{Q}}$ is total pulmonary blood flow, generally equal to cardiac output. We all know the O_2_Hb curve is not linear, but computer routines easily overcome the linear approximation by incorporating the actual dissociation curve when solving the equation. The value of using a linear dissociation curve approximation is considerable in that the result – (Equation 2) above – lays out clearly the three factors that dictate whether the diffusion process is complete (which would be the case if PecO_2_ reaches PAO_2_) or not (where PecO_2_ is less than PAO_2_). As can be seen, those factors are: DL, β, and $\dot{\text{Q}}$, coming together as the ratio DL/(β x $\dot{\text{Q}}$). The endcapillary PO2 can usually be assumed to equal that of systemic arterial blood, PaO_2_ (so that PecO_2_ in (Equation 2) = PaO_2_), and therefore (Equation 2) can be restated as:

(2) $$PAO_{2}-PecO_{2}=\left(PAO_{2}-PvO_{2}\right)\times \exp\left[-DL/\left(\beta \times \dot{Q}\right)\right]$$

Step 3: Perfusive transport in the arterial blood from lungs to muscle is of course accomplished by the heart as a pump delivering arterial blood via the systemic arterial tree. As with ventilation, perfusive transport is a ***convective*** process, with the blood circulating much like water flowing through a hose from high to low pressure. As with ventilation and gas movement, perfusion is powered by muscle contraction, but of course cardiac, not respiratory. Again in a steady state of O_2_ transport, mass conservation in perfusive transport can be expressed by a simple, well-known equation usually termed the Fick Principle:

(3) $$\dot{V}O_{2}=\dot{Q}\times \left[CaO_{2}-CvO_{2}\right]=\dot{Q}\times \beta \times \left[PaO_{2}-PvO_{2}\right]$$

$\dot{\text{Q}}$ is muscle blood flow while CaO_2_ and CvO_2_ are arterial and muscle venous blood [O_2_] respectively (also expressed as solubility, β, times partial pressure when approximating the O_2_ dissociation curve as linear). In words, the amount of O_2_ extracted from the blood and used by the muscle is the product of blood flow and arterio-venous [O_2_] difference. During heavy exercise in health, muscle blood flow takes the great majority of the cardiac output, and for conceptual simplicity, these are often, and here will be, taken to be numerically the same. Continuing the approximation of linearizing the O_2_ dissociation curve allows the substitution of partial pressures and dissociation curve slope (β) for concentrations as shown.

Step 4: Muscle microvascular to muscle mitochondrial diffusion constitutes the fourth step in the O_2_ transport pathway. As in the lung, this is a passive process (but in the red cell clearly in the opposite direction with O_2_ dissociating off Hb rather than combining with Hb as in the lung). After O_2_ dissociates from Hb it diffuses, dissolved in plasma, to and then through the vessel wall, interstitial fluid, muscle cell membrane and intracellular fluids to reach the mitochondria and the cytochromes within them. As in the lung, diffusion results in net movement of O_2_ from areas of higher PO_2_ (microvascular blood), to areas of lower PO_2_ (muscle mitochondria). A similar differential diffusion equation to that in the lung can be set up and integrated, and the result is this:

(4) $$PvO_{2}-PmO_{2}=\left(PaO_{2}-PmO_{2}\right)\times \exp\left[-DM/\left(\beta \times \dot{Q}\right)\right]$$

Here, PvO_2_ is muscle venous blood PO_2_, PmO_2_ is mitochondrial PO_2_, DM is muscle O_2_ diffusing capacity, and β and $\dot{\text{Q}}$ are as defined in (Equation 2) above. At peak exercise muscle is by far the largest contributor to mixed venous PO_2_. For simplicity, it is here assumed that muscle venous and mixed venous PO_2_ are numerically equivalent at peak exercise. This reflects the already-assumed equivalence of muscle and total blood flow stated above.

Step 5: Mitochondrial respiration is the fifth and final step in the O_2_ pathway. Technically, this “step” is not a transport process of moving O_2_ from point A to point B, making it a little different than the first four steps. Moreover, the process of mitochondrial respiration is a chemical process that follows the law of mass action. Through Michaelis-Menten kinetic analysis, mitochondrial respiration (also known as oxidative phosphorylation and describing muscle O_2_ utilization, $\dot{\text{V}}{\text{O}}_{2}$) can be shown to be a hyperbolic function of mitochondrial PO_2_ (PmO_2_) (Gnaiger, 2003; Scandurra and Gnaiger, 2010). The hyperbola is specified by it’s asymptote, often called Vmax, and its P_50_, the PO_2_ required to drive the mitochondria to half Vmax. Thus,

(5) $$\dot{V}O_{2}=Vmax\times PmO_{2}/\left[PmO_{2}+P_{50}\right]$$

This description of the O_2_ transport pathway thus includes 5 steps and each can be quantified by a single mass conservation-based steady-state equation. In a physiological and metabolic steady state, the values of PO_2_ at each step must simultaneously satisfy each of the equations in which they appear. This also means that wherever alveolar PO_2_ appears in the equations it has the same value in each equation. The same applies to arterial, venous and mitochondrial PO_2_’s. Similarly, $\dot{\text{V}}{\text{O}}_{2}$ in each equation must have the same value – precisely to conserve O_2_ mass. Thus the five equations form a simultaneous set (meaning that each dependent variable can take only one value across all steps in which they appear). Note also that the O_2_Hb dissociation curve (parametrized by its slope, β) appears in several of the equations. It has the same value in all equations in which it appears.

If the independent variables - ventilation (V̇E), cardiac output [or muscle blood flow ( $\dot{\text{Q}}$)], lung and muscle diffusing capacities (DL and DM respectively) and the O_2_Hb dissociation curve slope (β), are all measured at maximal exercise and mitochondrial Vmax and P_50_ are known [currently determined from muscle biopsy samples by *in vitro* respirometry (Gnaiger, 2003)], there remain 5 dependent variables spread across the 5 simultaneous equations: PAO_2_, PaO_2_, PvO_2_, PmO_2_ and $\dot{\text{V}}{\text{O}}_{2}$ itself. Solving these 5 equations thus provides a unique solution for the 5 dependent variables, and is straightforward by computer even when incorporating the actual O_2_ dissociation curve (rather than using its linear approximation) (Cano et al., 2013).

Thus the entire O_2_ transport system can be quantified from this equation set using the measured independent variables at peak exercise. In summary, the O_2_ partial pressures at every step (PAO_2_, PaO_2_, PvO_2_, PmO_2_) and $\dot{\text{V}}{\text{O}}_{2}$ itself are dependent variables which are determined by the values of the independent variables (Ventilation, blood flow, lung and muscle diffusing capacities, Hb dissociation curve characteristics and mitochondrial Vmax and P_50_). What is clear from these five equations is that by sharing variables and parameters, they functionally interact, meaning that how each step functions affects the function of the other steps. Said differently, each independent variable affects more than one step.

Perhaps the most easily understood example of this interaction is to consider what an increase in blood flow would do to the system: The most obvious and primary effect is, via step 3, an increase in O_2_ delivered from lung to muscle via the Fick Principle underlying that step. Thus, the muscle is offered more O_2_ per unit time as blood flow ( $\dot{\text{Q}}$) increases. However, a higher value for $\dot{\text{Q}}$ would impede diffusive equilibration and transport in both the lungs and muscle (steps 2 and 4) if it reduced time available for gas exchange, reflected by the presence of $\dot{\text{Q}}$ in the denominator of the exponent of the diffusion equations. This will partly offset the benefit of the greater convective delivery in step 3 (enabled by higher $\dot{\text{Q}}$). Just how much offset occurs would depend on how much the time available for gas exchange was actually reduced.

The point of this analysis is not to make the reader into a mathematical modeler. It is to convey an understanding of how O_2_ transport is structured and how it functions as an *integrated system* of tissues and organs that interact with (and thus affect) each other to set maximal $\dot{\text{V}}{\text{O}}_{2}$.

It also has the critical purpose of showing that there is no single determinant of $\dot{\text{V}}{\text{O}}_{2}\max$: All of the five steps and the factors underlying them mentioned above must individually and collectively contribute to $\dot{\text{V}}{\text{O}}_{2}\max$. There can be no one limiting factor to $\dot{\text{V}}{\text{O}}_{2}\max$. That will be the case whether at sea level or at altitude. How each independent variable would be expected to affect $\dot{\text{V}}{\text{O}}_{2}\max$ based on these equations has been explored (Wagner, 1996a) Overall, increases and modest decreases in any one of these variables have only small and similar directional effects on overall $\dot{\text{V}}{\text{O}}_{2}\max$ because of these interactions, while a large decrease in any one variable has a significant negative effect on $\dot{\text{V}}{\text{O}}_{2}\max$.

## Detailed interactions among the five pathway steps

As stated earlier, each pathway step is important in supporting $\dot{\text{V}}{\text{O}}_{2}\max$, but just as importantly, how the 5 steps interact with each other needs to be understood, with the example provided above of how changes in cardiac output have opposing effects on convective and diffusive transport. These interactions are best appreciated by noting that many of the variables involved appear in more than one of the mass conservation equations set out above. Thus, in terms of the five dependent variables (PAO_2_, PaO_2_, PvO_2_, PmO_2_ and $\dot{\text{V}}{\text{O}}_{2}$).

Equation 1 contains PAO_2_ and $\dot{\text{V}}{\text{O}}_{2}$.

Equation 2 contains PAO_2_, PaO_2_, and PvO_2_.

Equation 3 contains $\dot{\text{V}}{\text{O}}_{2}$, CaO_2_(ie, PaO_2_ x β) and CvO_2_ (ie, PvO_2_ x β).

Equation 4 contains PaO_2_, PvO_2_ and PmO_2_.

Equation 5 contains $\dot{\text{V}}{\text{O}}_{2}$ and PmO_2_ of mitochondrial respiration.

In tabular form, which includes the independent transport variables as well, these appearances are depicted in **Table 1** which may be easier to assimilate.

**Table 1:** Explicit appearance of independent (red)and dependent (blue) variables in each of the 5 O_2_ transport equations presented in the text.

Eqn	$\dot{\text{V}}\text{E}$	$\dot{\text{Q}}$	O_2_Hb dissn curve	DL	DM	Vmax & P_50_	$\dot{\text{V}}{\text{O}}_{2}$	PAO_2_	PaO_2_	PvO_2_	PmO_2_
1											
2											
3											
4											
5											

VE is ventilation; $\dot{\text{Q}}$ is cardiac output; O_2_ dissn curve is the slope of the O_2_Hb dissociation curve (β in the text); DL is lung diffusing capacity; DM is muscle diffusing capacity; Vmax and P_50_ are the asymptote and P_50_ of the mitochondrial respiration curve. The table shows multiple variables appearing in several equations. The message is that the 5 equations are not independent but inextricably linked as a system determining oxygen transport from the air to the mitochondria.

This table, depicting which equation includes which variables by the solid circles, clearly shows the many direct interactions among the components of the 5 equations through their appearance in multiple O_2_ pathway steps, and indicates that each independent variable (V̇E through Vmax and P_50_, red font in the table) affects more than one dependent variable ( $\dot{\text{V}}{\text{O}}_{2}$ through PmO_2_, blue font) and that dependent variables are directly affected by more than one independent variable.

**Table 1** has further implications: from the many combinations of their appearance in the equations, system performance overall ( $\dot{\text{V}}{\text{O}}_{2}$) is affected by each independent variable. In other words, despite direct effect of any one independent variable, there will be indirect effects seen via these interactions.

For example, **Table 1** shows that ventilation affects PAO_2_ directly (Equation 1). However, a change in PAO_2_ goes on to affect alveolar capillary diffusion (Equation 2). That in turn affects PaO_2_, which in turn affects PvO_2_ and hence $\dot{\text{V}}{\text{O}}_{2}$ itself (Wagner, 1996b). It also changes arterial PCO_2_ and hence pH (which changes the shape/slope of the O_2_Hb dissociation curve).

Similarly, cardiac output directly affects total O_2_ delivery from lungs to muscle, but at the same time affects both lung and muscle diffusion – and in the opposing direction. An increase in cardiac output works to raise arterial O_2_ delivery, but shortens red cell transit time in lungs and muscle, potentially reducing diffusive transport. A change in [Hb] has similar effects as for cardiac output: If [Hb] increases, arterial O_2_ delivery is raised, but via the diffusive processes (Equations 2, 4), through increasing β, impedes diffusive equilibration. A change in DL affects arterial PO_2_ and hence O_2_ delivery and in turn also PvO_2_. The same can be inferred for changes in DM. Changes in mitochondrial Vmax and P_50_ will affect PmO2 and/or $\dot{\text{V}}{\text{O}}_{2}$, which will induce changes in PaO_2_, PvO_2_ and PmO_2_ to keep the 5 equations solved with a unform set of PO_2_ values conserving O_2_ mass throughout the system.

This set of interactions seems impossibly complex to absorb, but with computers available to solve the five transport equations, the complicated ways in which all these interactions play out can be understood, especially using graphical analyses (Wagner, 1996a, 2023) and computer solutions to the five equations (Cano et al., 2013).

This lengthy analysis is felt essential to prepare the way to properly ask the question: What components of the system beyond the fall in PIO_2_ itself contribute to the loss of exercise capacity with altitude, and how? What physiological parts of the system are impacted by ascent to altitude? But first the experimental data on how much $\dot{\text{V}}{\text{O}}_{2}\max$ falls with altitude are presented.

## How much does $\dot{\text{V}}{\text{O}}_{2}\max$ fall with ascent to altitude?

There are several sources of this information, some from field studies in the mountains and some from altitude chamber studies. They all essentially agree, and so just two will be depicted **Figures 1**, **2**. These show the dramatic fall in $\dot{\text{V}}{\text{O}}_{2}\max$ in well-trained subjects participating in the American Medical Research Expedition to Everest (West et al., 1983) from >4.5 l/min to just 1 l/min at the Everest summit-altitude inspired PO_2_, and similar outcomes in Operation Everest II (OEII, Cymerman et al., 1989), which was conducted in an altitude chamber. For reference, a $\dot{\text{V}}{\text{O}}_{2}$ of 1 l/min supports only about 60 watts of external power, equivalent to a modest walk (perhaps 3 mph) on a level surface.

**Figure 1:**
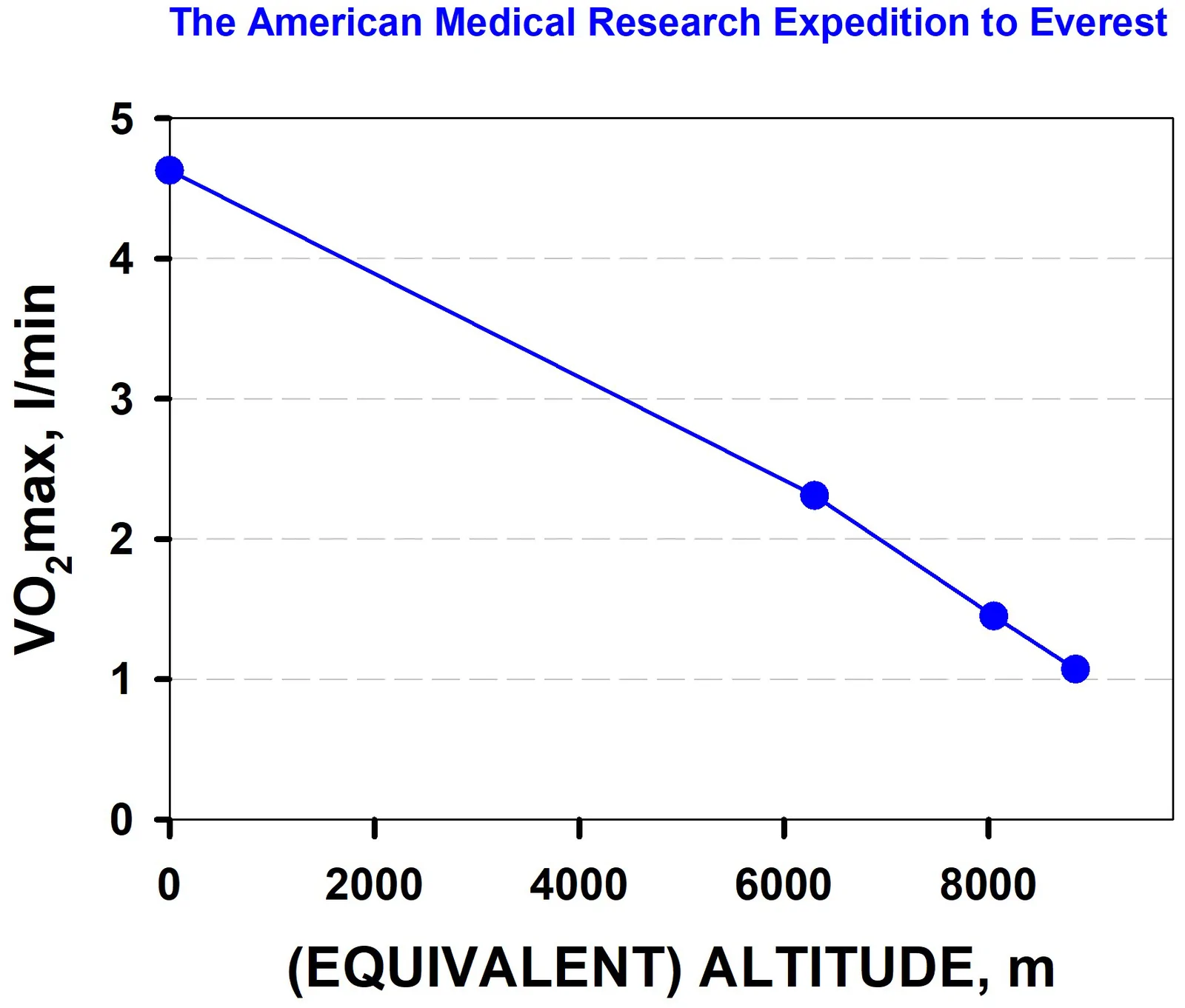
Fall in $\dot{\text{V}}{\text{O}}_{2}\max$ with altitude observed in the american medical research expedition to everest (AMREE) redrawn from data in West et al., 1983. There is a very large reduction from over 4.5 l/min at sea level to only about 1 l/min at the Everest summit PIO_2_.

## Reduction in $\dot{\text{V}}{\text{O}}_{2}\max$ with altitude just from the lower PIO_2_

The OEII study included invasive arterial and pulmonary arterial blood sampling, providing data to answer to the hypothetical question: what would $\dot{\text{V}}{\text{O}}_{2}\text{peak}$ have been just from the fall in PIO_2_ had there been *no* barriers to O_2_ transport and utilization. In particular, A) if there had been zero alveolar-arterial PO_2_ difference; B) if maximal cardiac output had remained as at sea level; and C) if all of the O_2_ in arterial blood had been extracted, moved to, and used by the mitochondria.

Answering A) requires calculating the expected arterial O_2_ saturation if arterial PO_2_ was not 28 mmHg, as measured at the Everest summit equivalent (Sutton et al, 1988), but equaled the alveolar PO_2_ (35 mmHg), eliminating the alveolar-arterial PO_2_ difference caused mainly by increased diffusive limitation in the lungs as shown by theoretical and experimental studies (West and Wagner, 1980; Torre-Bueno et al., 1985; Hammond et al., 1986; Wagner et al., 1986, 1987). Increasing arterial PO_2_ from 28 to 35 mmHg would have raised O_2_ saturation (to about 67%), 14 percentage points higher than the measured value of 53%. This relative increase of 26% would translate into a similar increase in arterial O_2_ concentration and O_2_ delivery.

Answering B) is straightforward. At sea level, in OEII, maximal cardiac output was 24 l/min while on the Everest summit equivalent it was only 16 l/min (Sutton et al., 1988). Restoring cardiac output from 16 to 24l/min would therefore afford a 50% increase in blood flow and O_2_ delivery to muscle. In acute hypoxia, which can be studied at up to about 15,000ft altitude, maximal cardiac output is the same as at sea level (Wagner et al., 1986; Mollard et al., 2007; Lehtonen et al., 2025), limiting the issue of reduced maximal cardiac output to higher altitudes, which also requires acclimatization.

Answering C) presumes the venous PO_2_ falls from 14 mmHg as measured at the Everest summit equivalent (Sutton et al., 1988) to zero, eliminating O_2_ diffusion limitation from blood to muscle. This would raise the arterio-venous O_2_ concentration difference, and thus $\dot{\text{V}}{\text{O}}_{2}$ as well, from 6.7 to 9.3 ml/dl, ie, by 39%.

As a consequence, $\dot{\text{V}}{\text{O}}_{2}\max$ would have been considerably higher than the observed value of 1.18l/min (Sutton J.R., et al), by 1.26 x 1.50 x 1.39, that is, by about 2.6 fold. Thus, on the Everest summit, without these O_2_ transport barriers, a $\dot{\text{V}}{\text{O}}_{2}$ max of about 3.1 l/min (1.18l/min x 2.6) would have been expected. This is shown in **Figure 2** (red symbols and line) with corresponding calculations at other altitudes.

**Figure 2:**
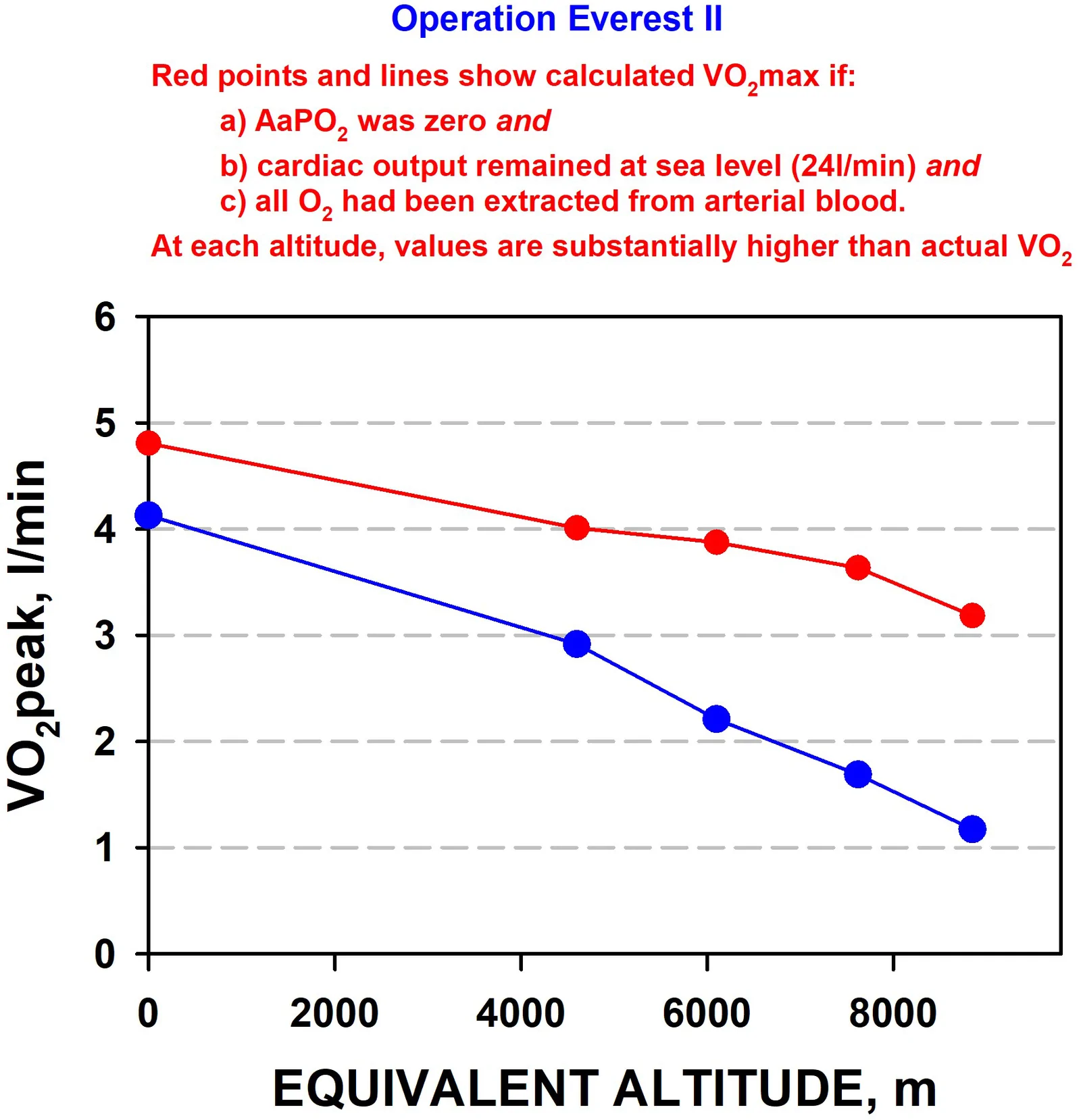
Fall in $\dot{\text{V}}{\text{O}}_{2}\max$ with altitude observed in operation everest II (redrawn and modified from Cymerman et al., 1989). There is a large reduction from 4.1 l/min at sea level to only 1.2 l/min at the Everest summit PIO_2_ (blue symbols), similar to that seen in AMREE. Red symbols show expected values of $\dot{\text{V}}{\text{O}}_{2}\max$ in the absence of barriers to O_2_ transport and utilization (ie, with maintained cardiac output, zero alveolar-arterial PO_2_ difference, and complete O_2_ extraction from arterial blood).

## Why is diffusion of O_2_ so vulnerable to altitude?

Equations 2, 4 provide the explanation, which follows from the O_2_Hb dissociation curve slope, β. At sea level, PaO_2_ during exercise in health is usually altered minimally (from at rest), positioned high on the flat portion of the O_2_Hb dissociation curve. Even as venous PO_2_ falls, sometimes to as low as 20 mmHg, the average slope of the O_2_Hb dissociation curve (β) at $\dot{\text{V}}{\text{O}}_{2}\max$ is relatively low, at about 0.2 ml O_2_/dl blood/mmHg. However, on the Everest summit equivalent, with arterial PO_2_ now at about 28 mmHg and mixed venous PO_2_ around14 mmHg (Sutton et al., 1988), gas exchange is occurring along the steepest portion of the O_2_Hb curve. Add in some polycythemia from time spent time at altitude (β is also proportional to [Hb]), and β is closer to 0.6. This roughly three-fold increase in β causes, via Equations 2 and 4, considerable alveolar-capillary and muscle O_2_ diffusion limitation during exercise. Note that PIO_2_ being around 43 mmHg on the Everest summit, (based on a barometric pressure of ~253 mmHg, West, 1999) also means that alveolar PO_2_ is low despite the high ventilation. Thus the driving gradient for alveolar-capillary diffusion – the difference between alveolar and lung capillary PO_2_ - is far less than at sea level. So too, the driving gradient between the blood and the mitochondria falls with altitude as arterial PO_2_ is reduced. The major influence of diffusion limitation on $\dot{\text{V}}{\text{O}}_{2}\max$ makes physical sense based on the ever-decreasing diffusion gradients for O_2_ with increasing altitude.

## Why might reduced cardiac output affect $\dot{\text{V}}{\text{O}}_{2}\max$ only minimally?

The argument against the reduction in peak cardiac output being a major factor in $\dot{\text{V}}{\text{O}}_{2}\max$ reduction is based mostly on theoretical considerations, and studies that increase cardiac output at altitude would be required to assess its actual effect on $\dot{\text{V}}{\text{O}}_{2}\max$. The most likely reason for lack of influence is that increasing cardiac output (eg., by 50% from the summit value of 16/l/min back to sea level values of 24l/min in OEII) could significantly reduce capillary transit time for red cells in both lungs and muscle, and thereby further increase diffusion limitation, offsetting much of the transport benefit enabled by the higher cardiac output (Wagner, 1996a). This reasoning presumes that in both lungs and muscles, capillary recruitment and distension are maximal at peak $\dot{\text{V}}{\text{O}}_{2}$ at all altitudes so that further flow increases could not raise capillary blood volume and thus buffer the effect of higher cardiac output on red cell transit time.

Indirect evidence supporting a minimal role for cardiac output affecting $\dot{\text{V}}{\text{O}}_{2}\max$ at altitude is that essentially restoring total O_2_ delivery to muscles via the polycythemia of altitude hardly alters $\dot{\text{V}}{\text{O}}_{2}\max$ (Calbet et al., 2002). While raising [Hb] is not the same as restoring cardiac output, it does restore overall arterial O_2_ delivery, the product of blood flow and O_2_ concentration. Based on the equations underlying O_2_ transport presented above, whether a given O_2_ delivery is accomplished by lower blood flow ( $\dot{\text{Q}}$) and higher [Hb] (ie, higher β) or any other combination of the two may not matter because their product - blood flow and β – is present together in both the diffusion equations (steps 2 and 4) and the convective equation (step 3). Thus, by inference from experimental studies in which [Hb] is manipulated without effect on $\dot{\text{V}}{\text{O}}_{2}\max$ at altitude, it would be expected that manipulation of blood flow would also not significantly affect $\dot{\text{V}}{\text{O}}_{2}\max$. However, until one can experimentally increase maximal cardiac output at altitude, the question does remain to be directly answered.

It should be noted that OEII had a major focus on cardiac function at altitude, and with invasive methodology on hand, this was evaluated by more than one team using complementary methods. Venous return curves showed no impairment of the right atrial pressure versus flow relationship (Reeves et al., 1987). Electrocardiography showed no myocardial ischemia (Malconian et al., 1990). Echocardiography even revealed enhanced left ventricular function (Suarez et al., 1987).

Multidisciplinary evidence thus shows remarkable preservation of cardiac function even in the extreme hypoxia of the Everest summit. The reduction in peak cardiac output cannot therefore be attributed to impaired cardiac function, and a worthwhile research target would be understanding why peak cardiac output falls at altitude, which might then lead to methods of experimentally raising it.

## Ventilation and mitochondrial function

Ventilation (step 1) and mitochondrial function (step 5) have so far been absent from this discussion. The reason for dismissing ventilation as a factor in the reduced $\dot{\text{V}}{\text{O}}_{2}\max$ of altitude is that at peak exercise in extreme hypoxia, ventilation is similar to or even greater than at sea level. For example, in OEII, peak ventilation was 124 l/min at sea level but 184 l/min at the Everest summit equivalent while arterial PCO_2_ at peak exercise, about 35 mmHg at sea level, fell to about 10mmHg at the summit (Sutton et al., 1988).

The influence on $\dot{\text{V}}{\text{O}}_{2}\max$ of possible mitochondrial functional changes with altitude is much less well studied, being based on limited *in vitro* measurements from muscle biopsies at rest. In deer mice native to high altitude, mitochondrial O_2_ affinity is increased compared to that in low altitude mice (Dawson and Scott, 2022). This implies that a higher $\dot{\text{V}}{\text{O}}_{2}$ can be sustained in high-altitude than low-altitude mice at a given mitochondrial PO_2_. However, the effects of altitude on mitochondria may not be simply proportional to the altitude attained. It is known that above about 5,000m, inexorable muscle mass loss is common in humans, and long term human habitation does not occur. Thus, observations made below 5,000m may be quite different than those found above these altitudes. Levett et al. (2012) found that mitochondrial biogenesis was deactivated in subjects who spent several weeks at Everest base camp (>5,000m) and above that altitude (also see Morash et al., 2012). While the fundamental biochemical process of oxidative phosphorylation would not be expected to change such that maximal $\dot{\text{V}}{\text{O}}_{2}$ would continue to depend on mitochondrial PO_2_ via Michaelis-Menten kinetics, that relationship is affected by the concentrations of the other molecules involved in the oxidative phosphorylation process. Clarifying the effects of altitude on mitochondrial function, both below and above 5,000m, would seem to be an important research goal in altitude biology. This would have clear implications for understanding $\dot{\text{V}}{\text{O}}_{2}\max$ at altitude.

## Small versus large muscle mass $\dot{\text{V}}{\text{O}}_{2}\max$ responses to altitude

The analysis presented is intended to discuss how usual exercise (ie, large muscle mass exercise, that brings the person to maximal values of ventilation and cardiac output) – affects $\dot{\text{V}}{\text{O}}_{2}\max$ at altitude. While O_2_ transport during small muscle mass exercise (such arm or one-leg activity) is expected to follow the same mass-conservation rules as for large muscle mass exercise, there are important differences. Perhaps most obvious is that maximal cardiac output is not achieved. This affords the possibility of greater compensation in O_2_ delivery at altitude compared to large muscle mass exercise. Indeed, it was found that during small muscle mass exercise at 5200m, O_2_ delivery and $\dot{\text{V}}{\text{O}}_{2}\max$ were restored to sea level values (Calbet et al., 2009). Notably, arterial PO_2_ at 55mmHg was 10mmHg higher and the alveolar-arterial PO_2_ difference was equivalently lower than during large muscle mass exercise. This was possibly due to lower cardiac output (compared to large muscle mass exercise) prolonging gas exchange time in the lung, lessening lung diffusion limitation. The higher arterial PO_2_ also served to enhance blood to muscle O_2_ diffusion. A full discussion of small muscle mass exercise at altitude is however beyond the scope of this perspective.

## Fitness, age, race/ethnicity and sex and $\dot{\text{V}}{\text{O}}_{2}\max$ at altitude

Finally, this Perspective has considered $\dot{\text{V}}{\text{O}}_{2}\max$ at altitude ignoring differences that might be seen depending on fitness, age, race/ethnicity and sex. While it seems unlikely that these factors would qualitatively alter the basic mechanisms discussed herein, there may well be systematic quantitative differences. It is known that high fitness results in greater loss in $\dot{\text{V}}{\text{O}}_{2}\max$ with altitude (Young et al, 1985; Lawler et al., 1988; Robergs et al., 1998). The latter study also found that males experienced greater $\dot{\text{V}}{\text{O}}_{2}\max$ loss than females, but others (Wagner et al., 1979) did not see this. Also, it would be expected that to the extent age is associated with lower $\dot{\text{V}}{\text{O}}_{2}\max$, older people may see less absolute $\dot{\text{V}}{\text{O}}_{2}\max$ reduction (Furian et al., 2022). However, exploring effects of altitude on reduced $\dot{\text{V}}{\text{O}}_{2}\max$ as a function of fitness, age, race/ethnicity and sex in detail is beyond the scope of this article.

## Summary

Understanding of how $\dot{\text{V}}{\text{O}}_{2}\max$ declines with altitude is incomplete. This is mostly because key experimental strategies required to provide a full understanding have not yet been devised or implemented. Calculations show that $\dot{\text{V}}{\text{O}}_{2}\max$ in OEII participants on the Everest summit equivalent would have been, remarkably, ~3l/min without the three main O_2_ transport barriers of A) lung diffusion limitation, B) reduced maximal cardiac output and C) muscle diffusion limitation (ie, incomplete O_2_ extraction from blood). The fall in PIO_2_ alone therefore explains about half of the total fall in $\dot{\text{V}}{\text{O}}_{2}\max$, and the three transport barriers together account for the remainder (by relatively similar amounts), further reducing $\dot{\text{V}}{\text{O}}_{2}\max$ to just 1.2l/min.

All steps in the O_2_ transport pathway contribute to setting $\dot{\text{V}}{\text{O}}_{2}\max$. However, it is here suggested that changes in the convective steps in O_2_ transport (ventilation, circulation of blood) are unlikely to explain much of the fall in $\dot{\text{V}}{\text{O}}_{2}\max$, even if important experimental interventions are not yet available to be certain about this conclusion. Peak ventilation is if anything higher than at sea level. Polycythemia has been shown not to improve $\dot{\text{V}}{\text{O}}_{2}\max$ at altitude. While peak cardiac output is considerably reduced at the Everest summit equivalent, restoring it to sea level values would likely confer only modest overall benefit to $\dot{\text{V}}{\text{O}}_{2}\max$ because gas exchange time in the lungs and muscles would fall, worsening diffusion limitation in both locations and opposing the gains in convective O_2_ transport in the blood. This hypothesis however remains to be directly tested.

On the other hand, there is good experimental evidence (supported by unequivocal theoretical calculations and logic) that the two diffusive steps in O_2_ transport – in the lungs and in the muscles – are increasingly significant barriers to preserving O_2_ delivery and extraction as PIO_2_ falls. Especially important is that O_2_ exchange in the lungs and muscles occurs on ever steeper parts of the O_2_Hb dissociation curve with increasing altitude. This inherently impedes the rate of diffusive equilibration in lungs and muscles, by reducing the diffusion gradients, and the result is reduction in $\dot{\text{V}}{\text{O}}_{2}\max$.

## Data Availability

The original contributions presented in the study are included in the article/supplementary material. Further inquiries can be directed to the corresponding author.
